# Meeting the Challenge of the "Know-Do" Gap: Comment on "CIHR Health System Impact Fellows: Reflections on ‘Driving Change’ Within the Health System"

**DOI:** 10.15171/ijhpm.2019.37

**Published:** 2019-05-28

**Authors:** David J. Hunter

**Affiliations:** Newcastle University, Newcastle, UK.

**Keywords:** Embedded Researchers, Researchers in Residence, Evidence-Informed Policy Research, Impact

## Abstract

Bridging the ‘know-do’ gap is not new but considerably greater attention is being focused on the issue as governments and research funders seek to demonstrate value for money and impact on policy and practice. Initiatives like the Canadian Institutes of Health Research (CIHR) Health System Impact (HSI) Fellowship are therefore both timely and welcome. However, they confront major obstacles which, unless addressed, will result in such schemes remaining the exception and having limited impact. Context is everything and as long as universities and research funders privilege peer-reviewed journal papers and traditional measures of academic performance and success, novel schemes seeking to break down barriers between researchers and end users are likely to have limited appeal. Indeed, for some academics they risk being career limiting. The onus should be on universities to welcome greater diversity and nurture and value a range of academic researchers with different skills matched to the needs of applied health system research. One size does not fit all and adopting a horses for courses approach would go a long way to solving the conundrum facing higher education institutions. At the same time, researchers need to show greater humility and acknowledge that scientific evidence is only one factor shaping policy and practice. To help overcome a risk of ideology and opinion triumphing over evidence, attention should be devoted to encouraging citizens to get actively involved in research. Research funders also need to give higher priority to how policy can be made to stick if the ‘know-do’ gap is to be closed.

The Perspective on the Canadian Institutes of Health Research (CIHR) Heath System Impact (HSI) Fellowship scheme is timely.^1^ Once again it would appear that the CIHR is leading the way in the drive to strengthen evidence-informed policy and practice in health systems. While experiments with ‘researchers in residence’ and ‘embedded researchers’ are not entirely new,^2,3^ only now are they becoming a more familiar feature of the knowledge-to-practice lexicon.^4^ But, for the most part, such initiatives in this area remain the exception and tend to be confined to isolated examples rather than be seeded and adopted across a country or whole system.

A possible exception can be found in the schemes launched by the Scottish Collaboration for Public Health Research and Policy, located at the University of Edinburgh, UK, and described by McAteer et al in their commentary on bridging the gap between research and policy and practice drawing on their experience of Scotland and Canada.^5^ Some of the new schemes have been launched jointly with the National Health Service in Scotland although how far there is take up across the country is not known. Where the Canadian HSI Fellowship seems to break new ground is its ambition to bridge the ‘know-do’ gap at scale across a whole system with a clear focus on impact fellows and to systematise their disparate experiences through the framework devised.

Evaluating the impact of embedded researcher initiatives to assess their ability to achieve their potential as they mature is of critical importance. But, as McAteer et al caution, any such initiative must be consistently implemented for a few years at least in order to be able realistically to expect any impact on the gap.

Risks remain over whether such initiatives, however well-intentioned, can succeed on their own without other concerns being addressed. Context is everything and there are many entrenched forces conspiring to weaken the impact of schemes to develop better links between academic and non-academic partners. Perhaps most critical among these is briefly mentioned at the end of the paper by Sim et al when the authors state that ‘it is unresolved how the training fellowship will impact future academic success.’ This goes to the nub of the issue and its resolution will determine the ultimate fate of the HSI and other similar schemes.

Hitherto, research funders and universities in different jurisdictions seem unable to resolve the conundrum facing them, seemingly to want to have their cake and eat it. On the one hand, research impact is now deemed to be desirable if not essential while, on the other hand, those applying for research funds and/or seeking promotion or career in academia are, by and large, still judged primarily on their prowess in publishing papers in high impact peer review journals.^6^ In the United Kingdom, in an attempt to adjust the balance in favour of applied research that can demonstrate real impact, the Research Excellence Framework now places greater emphasis on knowledge to action in its methodology for assessing and financially rewarding research institutions. With the methodology still under development, together with the time lag between completing the research and assessing any impact that can be attributed to it as distinct from numerous other factors at play in a complex, fast-moving area, it is too early to judge the effects of the change on the existing incentive structure within which academics operate.

Meanwhile, until otherwise advised or forced to change its ways, it is fair to say that the sector remains biased in favour of peer-reviewed, high impact journal papers. This is despite the fact that, as Sim et al rightly point out, ‘peer-reviewed literature is not the standard by which decisions are made.’ While the outputs of the HSI Fellowship may include academic publications, a wider range of different sorts of outputs are desired if health system change is to occur. But how these will be judged and weighted remains to be seen.

There is also a need to appreciate the complexity of things which compete with evidence – it is one of the most important lessons one academic acknowledged having to learn when making the transition from university to working in government.^7^ The HSI Fellowship is aimed at changing the culture in both academia and health systems and fellows, who may be academics or health professionals, are trained to take up positions as future health system and policy leaders rather than axiomatically pursuing an academic career. However, there is scant discussion of the particular challenges facing those seeking to make the transition from research and academic inquiry to the worlds of health systems and policy-making. More first-hand ethnographic accounts from those who have turned from poacher (academic researcher) to gamekeeper (policy-maker) would offer valuable learning for others contemplating such a move. It may be that more radical and imaginative action is called for. Instead of expecting academic researchers to be all things in all situations, one way of resolving the tension prevalent in universities, especially among the elite institutions, would be to endorse and encourage a more diverse, though equally valued, academic research workforce. The reality is that while an exceptional few academics might be able to excel in all domains irrespective of whether it involves teaching, grant capture, publishing peer-reviewed papers, and producing research that has real impact, we should acknowledge that the skill set required for each of these tasks differs.

We can all point to those exceptional teachers who struggle to pull in major grants. We also know of researchers who can attract large and regular grants but who may not always be the best communicators when performing before a group of undergraduate students. Then there are those researchers who are not always best able to collaborate or communicate with policy-makers and practitioners, often lacking the skills set and emotional intelligence required. Given this diversity there would be merit in adopting a ‘horses for courses’ approach, in keeping with how complex adaptive systems operate. It would mean acknowledging that the research endeavour is similarly complex and demands a repertoire of skills not all of which are to be found in a single individual.

We should be offering a flexible range of research career paths which might include the following: enabling practitioners and policy-makers to carry out research as part of their career development, including being joint authors on papers; allowing academic researchers to spend time working in policy and practice settings with the option of continuing such embedded attachments or returning to a more traditional research career if desired. Universities should be places where diversity is not only embraced but positively encouraged rather than academics finding certain options ‘off limits’ because they are regarded as inferior or career limiting.

Sadly, performance management regimes, like the UK Research Excellence Framework and Teaching Excellence Framework systems, and an increasing reductionist style of managerialism that has permeated higher education institutions, all too often serve to prohibit imaginative thinking and reduce flexibility in respect of academics pursuing a mix of interests at different stages of their careers. If the HSI Fellowship can navigate and overcome such myopic forces then it will have succeeded and hold valuable lessons for others seeking to strengthen the co-production of research between academic researchers, policy-makers, and practitioners. But there are at least three further obstacles to be confronted which may be less amenable to reform. First, the research industry in many countries, despite being aware of and sympathetic to criticisms of its primary focus, continues to promote the *doing* of research rather than its uptake, or lack of, by those who might profit from its findings.

Second, no matter how successful the efforts to promote evidence-informed policy and practice may be through efforts to promote collaboration between researchers and end users, if the political culture or context is antithetical to evidence use, then it hardly matters what happens to research.^8^ Moreover, co-production or co-creation approaches are not panaceas and bring with them their own pressures and costs including causing conflict, consuming resources and leading to misunderstandings.^9,10^

At a time when evidence is all too readily ignored or dismissed and is trumped by ideology or uninformed opinion, there may be a larger challenge facing researchers which goes beyond initiatives like the HSI Fellowship, necessary though they are. This requires broadening the range of audiences at which research is targeted, notably engaging the public more directly and encouraging research/evidence literacy among citizens so they, too, can join the research enterprise at all stages from initial development of the research questions to the final analysis and communication of findings. If citizens are able to contribute as co-creators of knowledge and its use then the current scepticism of scientific study might give way to a more informed appreciation of its strengths and weaknesses. Currently, research funders in many countries request research applicants to state how they will involve the public in their studies but invariably the requirement is treated somewhat tokenistically as an inconvenient tick box task rather than as an integral part of the study design.

Third, there is a debate to be had about the type of applied research needed and most likely to influence policy and practice. A great deal of funded health research is descriptive of the problem and even where solutions are proposed, far less attention is devoted to the messy business of implementation and to understanding the enablers and barriers involved in making policy stick. Yet, in much of the current discourse around health system transformation in various countries in Europe and beyond, the central issues are not around the Why or What questions but the *How* question, namely, how can effective transformation interventions be made to stick? Using best evidence is important in getting the policy right but unless there is also evidence of a readiness to change and the essential components of a receptive context for that change are in place, then the chances are that the ‘know-do’ gap to which Sim et al refer, and which is the trigger for the HSI Fellowship, will remain or even widen.^11,12^

Finally, there is a need for a greater degree of humility among all interested parties when considering the topic of evidence-informed policy and practice. A welcome acknowledgement of this is the shift in language away from evidence-*based* policy and practice. The framing is important because however much researchers may regret their lack of impact, the reality is that evidence from scientific inquiry is only one type of knowledge informing policy and not always the most critical.^13^ However, as this commentary has suggested, this does not mean that there is nothing to be done to strengthen the value and impact of research if proper and meaningful attention is given to the needs of end users and to the skill set required by those academics engaged in applied research. At the same time, hard-pressed policy-makers and practitioners seeking rapid and real-time research findings to demonstrate impact need to show a greater degree of realism about what value research can add and over a reasonable time frame. The temporal challenge is especially acute given the familiar research funding timescale of three to 5 years at the same time as the pace of policy-making is quickening and the impatience for evidence of what works grows. Locating that optimal balance can only come from a closer dialogue between the different parties concerned and if the HSI Fellowship, and similar schemes elsewhere, can facilitate this then it deserves to succeed and be supported.

## Ethical issues

Not applicable.

## Competing interests

Author declares that he has no competing interests.

## Author’s contribution

DJH is the single author of the paper.
